# Pleural Tuberculosis in Patients with Early HIV Infection Is Associated with Increased TNF-Alpha Expression and Necrosis in Granulomas

**DOI:** 10.1371/journal.pone.0004228

**Published:** 2009-01-19

**Authors:** Juanita Bezuidenhout, Teri Roberts, Liesel Muller, Paul van Helden, Gerhard Walzl

**Affiliations:** 1 Department of Histopathology, Tygerberg Hospital, Cape Town, Western Cape, South Africa; 2 Molecular Biology and Human Genetics, MRC Centre for Molecular and Cellular Biology, DST/NRF Centre of Excellence for Biomedical TB Research, Department of Biomedical Sciences, Faculty of Health Sciences, University of Stellenbosch, Cape Town, Western Cape, South Africa; University of Stellenbosch, South Africa

## Abstract

Although granulomas may be an essential host response against persistent antigens, they are also associated with immunopathology. We investigated whether HIV co-infection affects histopathological appearance and cytokine profiles of pleural granulomas in patients with active pleural tuberculosis (TB). Granulomas were investigated in pleural biopsies from HIV positive and negative TB pleuritis patients. Granulomas were characterised as necrotic or non-necrotic, graded histologically and investigated for the mRNA expression of IL-12, IFN-γ, TNF-α and IL-4 by *in situ* hybridisation. In all TB patients a mixed Th1/Th2 profile was noted. Necrotic granulomas were more evident in HIV positive patients with a clear association between TNF-α and necrosis. This study demonstrates immune dysregulation which may include TNF-α-mediated immunopathology at the site of disease in HIV infected pleural TB patients.

## Introduction

Granulomas represent a distinctive pattern of chronic inflammation that involves adaptive immunity, and are the hallmark of many human diseases of significance, such as tuberculosis (TB) [1], [2]. An important gap in current knowledge is an understanding of how host resistance to TB disease is mediated. The pleura is often involved in active TB disease and this can take on the form of effusions, tuberculous empyema or obliterative fibrous pleuritis [3]. The release of mycobacterial antigens into the pleural space, eliciting a delayed-type hypersensitivity (DTH) reaction [4], may play an important role in the pathogenesis of tuberculous pleurisy. Pleural TB in human immunodeficiency virus (HIV)-negative subjects has been taken to represent more of a protective immune response to *Mycobacterium tuberculosis* (*Mtb*) than other forms of the disease due to the fact that a high proportion of individuals can recover without antibiotic therapy [5].

Granuloma formation is considered to be an essential component of protective immune responses against *Mtb* [6]. The formation of the granuloma is firmly linked to the DTH reaction, which is a form of adaptive cell-mediated immunity (CMI) that is mediated by activated T lymphocytes and their cytokines. Epithelioid macrophages are present in the centre of the granuloma with some multi-nucleated giant cells and this central area is surrounded by lymphocytes. Dendritic cells may also be present.

HIV infected people develop TB at a rate of 10% per year, rather than the norm of 10% per lifetime [7], [8], suggesting that HIV infection may affect the ability of granulomas to contain *Mtb*. It has been hypothesized that quantitative and qualitative T-helper responses may impact on AIDS pathogenesis. Although the supposed role of modulation of T helper responses in HIV pathogenesis remains controversial, a switch in the predominant response from Th1 to Th2 and the production of associated cytokines may facilitate disease progression [9], [10], [11]. HIV coinfection weakens the granulomatous host response to *Mtb* due to insufficient macrophage, T cell and cytokine responses [12], [13], [14], [15]. TB is often an early occurrence in HIV, even before any reduction in CD4+ T cell count [16], and the trafficking of HIV infected, and therefore dysfunctional, cells into the granuloma may lead to granuloma disruption. *Mtb* can also modulate HIV replication by generating a cytokine microenvironment favouring viral infection and spread in local mononuclear cells [17], [18].

There is a delicate and complicated interplay between different cytokines and there are numerous reports of beneficial and detrimental effects of individual cytokines. High levels of pro-inflammatory cytokines, including IFN-γ and TNF-α [19] are found in TB effusions but, in addition to protective effects [20], [21], there are also reports of detrimental effects of IFN-γ [22], TNF-α [23], and IL-12 [24], especially if present at high concentrations. The role of IL-4 in the immunopathology of TB [25], [26] has been established and may be due to the suppression of the Th1 cytokines IL-12, IFN-γ and TNF-α.

Understanding the human host response is important, as this would assist in the development of new vaccines and immunomodulatory adjuvants for the prevention or treatment of TB. We chose to investigate the Th1/Th2 profile in pleural TB patients by measuring the mRNA expression levels of the cytokines IL-12, IFN-γ, TNF-α and IL-4 *in situ*. In addition we compared the cytokine profiles of necrotic and non-necrotic granulomas from HIV-positive and -negative patients in order to describe the association between cytokine production, necrosis and HIV positivity.

## Materials and Methods

### Study design, participants and tissue specimens

A total of 12 patients with pleural TB were recruited from Tygerberg Academic Hospital, including 6 HIV sero-negative (group 1) and 6 HIV sero-positive (group 2) people. HIV serology was done on all patients by ELISA. HIV positive patients were in the early stage of the disease and had no opportunistic infections besides TB. All patients presented with symptoms of pleuritis and had radiological confirmation of the effusion. None of the patients had had TB previously nor were any on treatment at the time of the study. Pleural biopsy tissue was obtained for diagnostic purposes. The diagnosis of TB was confirmed by the presence of granulomatous inflammation and Ziehl-Neelsen (ZN) positive bacilli in biopsy material [27]. Standard, fixed-dose anti-TB treatment was administered before the patients were referred to TB clinics. No follow-up data on these patients was available. Approval was granted by the Ethics Committee of the University of Stellenbosch. Written, informed consent was obtained from all the participants.

### Preparation of Riboprobes

This was performed essentially as described previously [28], [29] using PCR conditions and primer sequences as published elsewhere [30], [31]. Briefly, peripheral blood mononuclear cells (PBMCs) were isolated from a healthy volunteer and stimulated for 18 hours with phytohemagglutinin (PHA). Total RNA was extracted and quantified and shown to be undegraded on a 1% agarose gel. cDNA was prepared using the Titan system (Boehringer Mannheim). The PCR products were cloned into the vector pGEM7Zf (Promega) and sequencing of the clones confirmed the DNA sequence (Table 1) and ascertained the orientation of the PCR product in order to synthesize sense and antisense riboprobes. After digestion the fragments were separated by electrophoresis on 1% agarose gels and extracted. RNA polymerases were used to transcribe digoxigenin-labelled RNA and labelling of the probes was confirmed by Northern blot analysis.

**Table 1:** Sequences of cytokine riboprobes used in *in situ* hybridisation.

Riboprobe	Length (bp)	Sequence (5′ – 3′)
TNF-α	124	TCTCGAACCCCGAGTGACAAGCCTGTAGCCCATGTTGTAGCAAACCCTCAAGCTGAGGGGCAGCTCCAGTGGCTGAACCGCCGGGCCAATGCCCTCCTGGCCAATGGTGTGGAGCTGAGAGATA
IFN-γ	356	AGTTATATCTTGGCTTTTGAGCTCTGCATCGTTTTGGGTTCTCTTGGCTGTTACTGCCAGGACCCATATGTACAAGAAGCAGAAAACCTTAAGAAATATTTTAATGCAGGTCATTCAGATGTAGCGGATAATGGAACTCTTTTCTTAGGCATTTTGAAGAATTGGAAAGAGGAGAGTGACAGAAAAATAATGCAGAGCCAAATTGTCTCCTTTTACTTCAAACTTTTTAAAAACTTTAAAGATGACCAGAGCATCCAAAAGAGTGTGGAGACCATCAAGGAAGACATGAATGTCAAGTTTTTCAATAGCAACAAAAAGAAACGAGATGACTTCGAAAAGCTGACTAATTATTCGGT
IL-4	317	CTTCCCCCTCTGTTCTTCCTGCTAGCATGTGCCGGCAACTTTGTCCACGGACACAAGTGCGATATCACCTTACAGGAGATCATCAAAACTTTGAACAGCCTCACAGAGCAGAAGACTTGTGCACCGAGTTGACCGTAACAGACATCTTTGCTGCCTCCAAGAACACAACTGAGAAGGAAACCTTCTGCAGGGCTGCGACTGTGCTCCGGCAGTTCTACAGCCACCATGAGAAGGACACTCGCTGCCTGGGTGCGACTGCACAGCAGTTCCACAGGCACAAGCAGCTGATCCGATTCCTGAAACGGCTCGACAGGAA
IL-12p40	301	CCAACAACTTGCAGCTGAAGCCATTAAAGAATTCTCGGCAGGTGGGAGTACCCTGACACCTGGAGTACTCCACATTCCTACTTCTCCCTGACAGGTCCAGGGCAAGAGCAAGAGAGAAAAGAAAGATAGAGTCTTCACCGACCACGGTCATCTGCCGCAAAAATGCCAGCATTAGCGTGCGGGCCCAGGACCTCATCTTGGAGCGAATGGGCATCTGTGCCCTGCAGTTAGGTTCTGATCTTTGGAGGAAAAGTGGAAGATATTAAGCAAAATGTTTAAAAGACACAACGGAATAGACCCA

### RNA-RNA *In Situ* Hybridisation and Dual Labelling

This was performed essentially as described previously [28], [29] on paraffin-embedded lung tissue.

Briefly, consecutive 5 µm sections were applied to RNase-free slides previously coated with aminopropyltriethoxysilane (5 μg/ml, Sigma Aldrich), deparaffinized in xylene, rehydrated through graded ethanols and diethyl pyrocarbonate-treated water and incubated in phosphate-buffered saline (PBS). The sections were treated with proteinase K in Tris-HCl-EDTA, washed with PBS, refixed in paraformaldehyde and acetylated in triethanolamine-acetic anhydride. The slides were then rinsed, dehydrated and air-dried before hybridization. Sections were incubated in a mixture of dextran sulphate, Tris-HCl, Denhardt's solution, EDTA, dithiothreitol, herring sperm DNA, tRNA and deionized formamide. Sections were hybridized for 18 hours in a humidified chamber at 50°C, washed in saline sodium citrate, incubated in Tris-HCl-NaCl containing sheep serum and then washed. Thereafter, sections were incubated with antidigoxigenin antibody conjugated to alkyline phosphatase (Boehringer Mannheim) and the signal detected with BCIP-NBT-INT (Dako). After the appearance of a brown colour slides were counterstained with Mayer's hematoxylin (Sigma Aldrich), rinsed in distilled water, mounted with Dako Faramount and viewed under a light microscope.

For dual labelling, slides were rinsed and non-specific protein blocked with milk powder and Triton-X 100 in PBS. Sections were incubated with antibody diluted in goat serum, washed and labelled with secondary biotinylated goat anti-mouse antibody diluted in goat serum and Triton-X 100. Sections were washed again, incubated with streptavidin conjugated to alkyline phosphatase, washed in PBS and incubated with a solution of fast red (Vector Laboratories). Slides were counterstained with hematoxylin and mounted with Dako Faramount.

### Photography and Assessment of Slides

**Figure 1:**
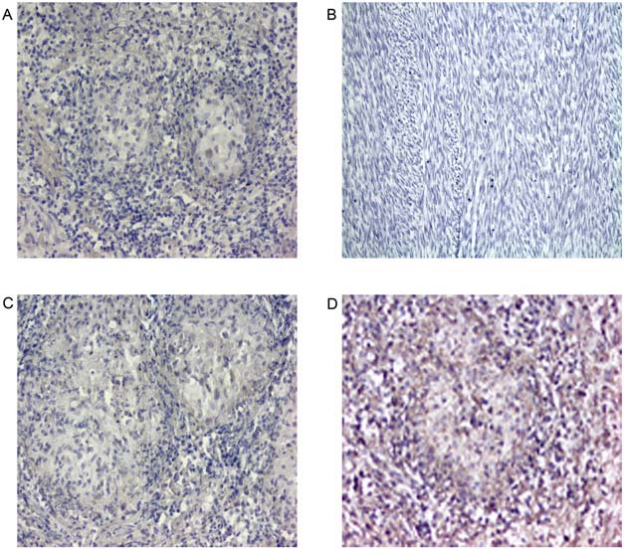
Positive and negative control staining in human tissue. Paraffin-embedded tissue was stained using ISH for mRNA expression. Negative and sense probe controls were repeated each time lung tissue was stained for cytokine expression. In the first control (A) lung granulomatous tissue was not stained with probe. The absence of brown staining proves that there was no nonspecific staining. Magnification 200×. In the second control (B) anti-sense probe was applied to tissue known not to produce cytokines (sarcoma). The absence of brown staining proves that there was no nonspecific staining. Magnification 400×. In the third control (C) granulomatous lung tissue was stained with sense probe. The absence of brown staining represents specificity. Magnification 200×. In the last control (D) granulomatous lung tissue was stained with the anti-sense β-actin probe. Brown staining indicates the presence of non-degraded mRNA. β-actin expression is diffusely positive. Magnification 200×.

**Figure 2:**
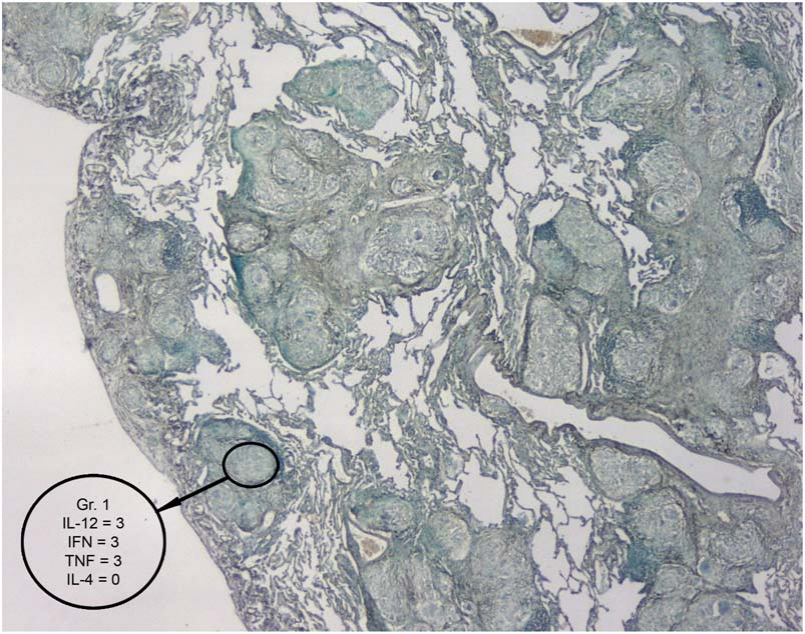
A granuloma map used to assess the slides from patients with granumatous diseases. This example represents the type of granuloma map used to assess the tissue biopsies of patients with pleural TB for the absence or presence of granulomas and type (necrotic or non-necrotic) of granuloma. Once the granuloma was identified, ISH was used to stain the tissue for mRNA cytokine expression. Magnification 25×.

Images were captured using a Zeiss light microscope with light parameters optimized for the actin stained slide. These settings were kept constant in order to maintain comparability between slides. Control slides were added parallel to each staining procedure to show that the positive colour was specific and that the mRNA was not degraded (Figure 1). The number of necrotic and non-necrotic granulomas were determined by H+E stain. A map of each section of the granuloma was created by photographing the slides at ×25 magnification (Figure 2). Each granuloma was subsequently numbered. The slides were then examined under the light microscope and the cytokine profile of each numbered granuloma was graded (at ×400 magnification) on the photographic map as follows:

- >75% of cells in a granuloma positive: grade 3 positivity
- 25% to 75% of cells in a granuloma positive: grade 2 positivity
- <25% of cells in a granuloma positive: grade 1 positivity
- no cells positive: grade 0 positivity

### Statistical analysis

All granulomas were regarded as independent parameters.

Statistical analysis on samples from patients with pleural TB was performed by applying the Pearson Chi square and Spearman rank R tests to the following parameters:

- The association between HIV serology and presence of necrosis.
- The association between HIV and individual cytokines
- Comparisons in association patterns between various cytokines between HIV negative and HIV positive granulomas.
- The association between necrosis and individual cytokines.
- Comparisons in association patterns between various cytokines between necrotic and non-necrotic granulomas.

In addition, to test for association between pairs of cytokines and necrosis a two-tailed Fisher's exact test was used.

## Results

### Demographic and clinical data

There were 2 male and 4 female TB patients in the HIV positive group with a mean age of 34.5 years compared to 5 males and 1 female with a mean age of 38 years in the HIV negative group. All patients presented with symptoms of pleuritis. All pleural biopsies were subsequently found to be culture positive for drug-sensitive *Mtb*.

### Necrosis is more prevalent in granulomas of HIV positive patients

**Figure 3:**
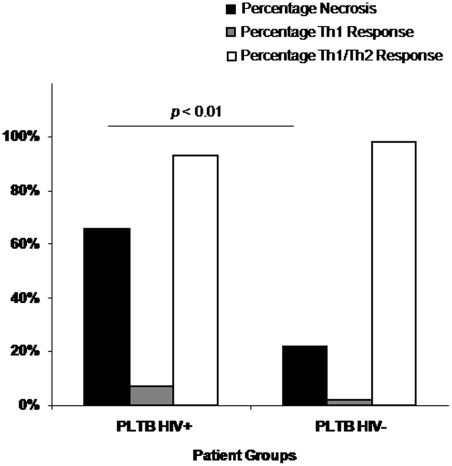
Necrosis and expression of mixed Th1/Th2 and Th1 responses in granulomas of pleural tuberculosis patients. The percentage of granulomas with necrosis and expression of Th1 and Th2 cytokines were assessed microscopically. Groups include HIV positive (PLTB HIV+, n = 6) and HIV negative (PLTB HIV−, n = 6) pleural TB patients (n = 12). The percentage of necrosis is represented by solid bars, the percentage of Th1 response by clear bars and the percentage of a mixed Th1/Th2 response by striped bars. There was a significantly higher percentage of necrotic granulomas in HIV positive compared to HIV negative TB patients (Bootstrap test *p*<0.01).

Of the 166 granulomas present in pleural biopsies from the 12 patients with pleural TB, 125 were from HIV negative patients and 41 from HIV positive patients. Necrosis was more prevalent in granulomas of HIV positive patients (Figure 3). In the 6 HIV positive patients, 27 (66%) of granulomas were necrotic whereas only 21 (17%) were necrotic in the HIV negative group (Chi-square test *p*<0.01).

### Cytokine expression is upregulated in necrotic granulomas and granulomas of HIV positive TB patients

**Figure 4:**
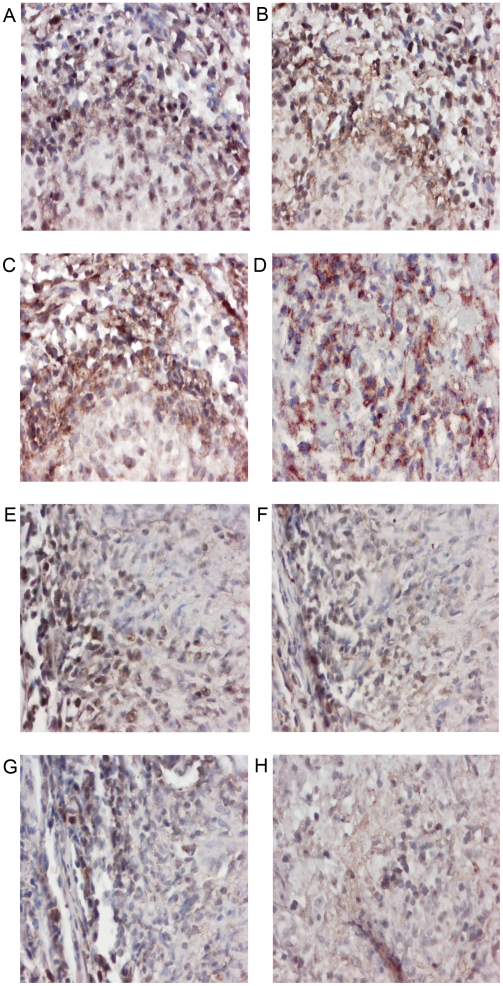
Cytokine mRNA expression in HIV positive and negative pleural tuberculous granulomas. Paraffin-embedded tissue was stained using ISH for mRNA expression. Representative sections of pleural needle biopsies from HIV positive (A–D) patients are shown. (A) Staining with the anti-sense IL-12 probe shows that over 75% of cells are positive for IL-12 mRNA expression. Magnification 400×. (B) Staining with the anti-sense IFN-γ probe shows that over 75% of cells are positive for IFN-γ mRNA expression. Magnification 400×. (C) Staining with the anti-sense TNF-α probe shows that over 75% of cells are positive for TNF-α mRNA expression. Magnification 400×. (D) Staining with the anti-sense IL-4 probe shows that over 75% of cells are positive for IL-4 mRNA expression. Magnification 400×. Representative sections of pleural needle biopsies from HIV negative (E–H) patients are shown. (E) Staining with the anti-sense IL-12 probe shows that 25%–75% of cells are positive for IL-12 mRNA expression. Magnification 100×. (F) Staining with the anti-sense IFN-γ probe shows that 25%–75% of cells are positive for IFN-γ mRNA expression. Magnification 100×. (G) Staining with the anti-sense TNF-α probe shows that 25%–75% of cells are positive for TNF-α mRNA expression. Magnification 100×. (H) Staining with the anti-sense IL-4 probe shows that less than 25% of cells are positive for IL-4 mRNA expression. Magnification 100×.

Pleural biopsy tissue was examined for IL-12, IFN-γ, TNF-α and IL-4 mRNA expression using *in situ* hybridization (ISH). Representative images illustrate the presence of both Th1 and Th2 cytokine mRNA expression, all of which were more prevalent in necrotic granulomas (Figure 4). The extent of Th1 and mixed Th1/Th2 responses was not different between granulomas of HIV positive and negative TB patients (Figure 3).

While the presence of necrosis did not significantly influence the grade of IL-12 mRNA positivity, IFN-γ, TNF-α and IL-4 mRNA expression were markedly upregulated in necrotic granulomas (Table 2). Similarly, IL-12, IFN-γ, TNF-α and IL-4 mRNA expressions were markedly upregulated in both necrotic and non-necrotic granulomas of HIV positive TB patients (Table 3).

**Table 2:** The percentage of necrotic and non-necrotic granulomas positive for specific cytokines in patients with pleural tuberculosis.

	Cytokine expression grade Non-necrotic granulomas				Cytokine expression grade Necrotic granulomas				Chi-square test
	0	1	2	3	0	1	2	3	
IL-12	0	31%	44%	25%	0	23%	54%	23%	*p* = 0.50
IFN-γ	0	48%	35%	17%	0	35%	25%	40%	*p* = 0.01
TNF-α	0	35%	42%	24%	0	17%	19%	64%	*p*<0.01
IL-4	3%	50%	26%	21%	6%	17%	42%	35%	*p*<0.01

The presence of IL-12, IFN-γ, TNF-α and IL-4 positive cells was assessed by *in situ* hybridisation in granulomas from pleural biopsies. Positivity was graded according to the total number of cells staining positive in a specific granuloma (<25%, grade 1; 25–75%, grade 2 and >75%, grade 3).

**Table 3:** The percentage of granulomas positive for specific cytokines in HIV negative and HIV positive pleural tuberculosis patients.

	Cytokine expression grade HIV negative granulomas				Cytokine expression grade HIV positive granulomas				Chi-square test
	0	1	2	3	0	1	2	3	
IL-12	0	31%	46%	23%	0	20%	49%	31%	*p* = 0.03
IFNγ	0	50%	30%	20%	0	26%	37%	37%	*p* = 0.02
TNFα	0	31%	42%	27%	0	24%	15%	61%	*p*<0.01
IL-4	2%	46%	26%	26%	7%	24%	46%	23%	*p* = 0.02

IL-12, IFN-γ, TNF-α and IL-4 mRNA expression was examined by *in situ* hybridisation on granulomas from pleural biopsies. Positivity was graded according to the total number of cells staining positive in a specific granuloma (<25%, grade 1; 25–75%, grade 2 and >75%, grade 3).

### Cytokine expression in necrotic granulomas and granulomas of HIV positive TB patients is dysregulated

There was a strong positive correlation between all cytokines expressed in non-necrotic granulomas and granulomas from HIV negative TB patients (Table 4). By contrast, expression of fewer cytokines in necrotic granulomas were correlated and cytokines expressed in granulomas of HIV positive TB patients were not correlated at all, with the exception of IL-12 and TNF-α whose expression was negatively correlated. It is interesting to note that IL-4 expression, representing a Th2 response, was correlated to the expressions of the Th1 cytokines IL-12, IFN-γ and TNF-α.

**Table 4:** Spearman rank R correlation for cytokines in granulomas of pleural TB patients.

Cytokines	Type of Granuloma			
	Non-necrotic	Necrotic	HIV negative	HIV positive
IL-12 vs IFN-γ	0.49 (0.00)*	0.32 (0.83)	0.49 (0.00)*	−0.16 (0.30)
IL-12 vs TNF-α	0.44 (0.00)*	−0.17 (0.24)	0.49 (0.00)*	−0.45 (0.003)*
IL-12 vs IL-4	0.39 (0.00)*	0.21 (0.15)	0.47 (0.00)*	−0.04 (0.80)
IFN-γ vs TNF-α	0.58 (0.00)*	0.63 (0.00)*	0.66 (0.00)*	0.38 (0.01)
IFN-γ vs IL-4	0.33 (0.0003)*	0.65 (0.00)*	0.52 (0.00)*	0.22 (0.16)
TNF-α vs IL-4	0.46 (0.00)*	0.34 (0.02)*	0.59 (0.00)*	0.08 (0.60)

IL-12, IFN-γ, TNF-α and IL-4 mRNA expression was examined by *in situ* hybridisation on granulomas from pleural biopsies. Statistically significant correlations are noted with * with *p*-values in brackets.

## Discussion

This work characterised cytokine expression at the site of disease during the granulomatous response in pleural TB, in HIV infected and uninfected individuals and in necrotic and non-necrotic granulomas. Patients with pleural TB presented with a mixed Th1/Th2 profile and this contradicts a characterisation of HIV negative pleural TB as Th1 dominant [32]. The main findings were a strong presence of TNF-α positive cells in necrotic granulomas, particularly in granulomas from HIV positive patients, where increased necrosis has previously been described [33]. Although we found high levels of TNF-α in our HIV positive TB patients, Noronha *et al* found the opposite [34]. This discrepancy may be due to different stages of immunosuppression in the two studies. In the present study granuloma morphology was intact, which suggests relative immune competence, whereas poorly formed or absence of granulomas, as seen in the Noronha study, may indicate advanced immunosuppression [33]. However, the presence of high expression levels of TNF-α mRNA as seen in our study does not necessarily equate to increased presence of TNF-α protein and may even be representative of a failed attempt to induce cytokine production.

The investigation of immune responses at the site of disease is extremely valuable because cytokine responses do not necessarily mimic those found in the peripheral blood [19], [35]. This may be due to a redistribution of antigen specific cells to the site of disease (compartmentalization), where their effects on the eventual outcome of infection may be crucial.

Although the presence of TNF-α is beneficial in the immune response against *Mtb*, and is necessary for granuloma formation, it does cause immunopathology such as caseous necrosis [23], [36]. This is probably a consequence of apoptosis of infected macrophages, epithelioid cells and activated T cells within the granuloma [37], [38] as has been shown in human TB [37]. The link between the apoptotic activity of TNF-α [39], [40] and the formation of caseous necrosis may be due to secondary necrosis (reviewed in [41]). Whereas apoptosis is a strictly regulated process, and leads to the containment of cell debris, necrosis is unregulated and results in the spilling of intracellular contents. The processes may become linked when cell uptake is disrupted and the rate of cell death exceeds the rate at which macrophages are able to phagocytose apoptotic material. This leads to the impaired uptake of apoptotic cells and during this delay necrosis may develop. Thus, although primary necrosis may be very rare during lung inflammation due to *Mtb* infection, secondary necrosis may be common [42]. This mechanism may be compounded by the *Mtb*-mediated lysis of macrophages which triggers the macrophage cell death pathway. This not only allows dissemination of the bacilli but also contributes to the formation of necrotic lesions [43].

Although apoptosis is an important host defence mechanism to deprive *Mtb* of its intracellular sanctuary and inhibit its growth and survival [44], [45], it also facilitates mycobacterial dissemination and may therefore be orchestrated by the bacillus itself [46], [37]. It is unclear whether apoptosis favours the host or the pathogen as impaired apoptosis also maintains granulomas by preventing T cell clonal deletion [47]. The viability of *Mtb* after Fas ligand or TNF-α mediated apoptosis of immune cells is controversial with both reduced and unchanged bacterial numbers reported [48], [49].

Previous studies that investigated correlations between granuloma histology and cytokine mRNA expression [28], [29] suggested that the presence of IL-4 may not be an indicator of poor prognosis in pulmonary TB patients but may rather be an integral feature of tuberculous granuloma formation and may limit tissue damage. Their results showed that IL-4 positive granulomas tended to be non-necrotic whereas all necrotic granulomas were TNF-α positive and negative or weakly positive for IL-4 and IFN-γ. Although we cannot extrapolate from these findings to a causative relationship between TNF-α and necrosis, it appears likely that TNF-α may promote necrosis. The caseous necrosis appears to be a consequence of apoptosis of infected macrophages and activated T cells and TNF-α is a known inducer of apoptosis [39]. An essential role for IL-12 and IFN-γ in protective immunity to mycobacterial infection has been previously described [50]. Furthermore individuals with defective IL-12 or IFN-γ receptors are susceptible to disseminated mycobacterial infection [51] and decreased IFN-γ production by peripheral blood mononuclear leukocytes has been shown to correlate with decreased IL-12 receptor subunits B1 and B2 in patients with tuberculosis [52].

The cytokine pattern observed in patients with pleural TB reflects a model by Wigginton and Kirscher [53] that was designed to predict CMI during human infection with *Mtb*. We found increased IFN-γ expression in necrotic granulomas and, although, like TNF-α, it is crucial in defence against *Mtb*, high effector cytokine levels during chronic phases of disease may contribute to immunopathology, especially in the presence of high TNF-α expression [54], [36], [53]. The strong correlation between TNF-α and IFN-γ expression and HIV positivity supports the concept that cytokine overproduction may represent an attempt to compensate for the failure of other CD4 effector T cell functions, including proper granuloma formation and the antigen-specific activation of macrophages [19], [55], [53].

By contrast, non-necrotic granulomas with a high number of IL-12 positive cells also expressed high levels of IL-4 and this was not associated with necrosis presumably because Th2 cytokines mediate local tissue inflammation and necrosis through their effect on TNF-α induced cytotoxicity [56], [57]. IL-4 may therefore provide a regulatory mechanism to antagonise necrosis-inducing factors but in doing so may also shift infection from latency to disease [53]. This is in contrast to other studies which have found that IL-4 works in synergy with TNF-α to increase necrosis (reviewed in [58]). We did not measure IL-4's natural antagonistic splice variant, IL-4δ2, which has been found to be expressed in the lungs of TB and TB-HIV co-infected patients [59], [60]. It is therefore possible that the upregulation of IL-4 seen in non-necrotic granulomas may be due, in part, to an upregulation of the splice variant. However, we still believe that IL-4 plays a predominant role because an upregulation of the splice variant would have facilitated an increase in inflammation and necrosis. Dheda *et al* [60] have also highlighted that, during infection with HIV, the switch from a Th1 to a Th2 response may not necessary be responsible for the progression to AIDS in the absence of opportunistic infections. However, when co-infected with TB, IL-4 expression is upregulated and this may allow an increase in viral replication.

Interestingly very few necrotic granulomas expressed a high level of IFN-γ-positive cells in the presence of high IL-12 expression. This may suggest a breakdown in the regulatory function of IL-12 which usually regulates the expression of IFN-γ [61], [62]. Non-necrotic granulomas tended to express low levels of all cytokines which suggests that a lower cytokine presence is less destructive.

In summary, intracellular bacteria that can survive within macrophages lead to granuloma formation, which benefits the host by controlling local and systemic dissemination of pathogens but facilitates destructive pulmonary pathology. In TB, this manifests as caseous necrosis with tissue destruction and subsequent fibrosis. This facilitates disease transmission and therefore blocking any mechanism responsible for necrosis would also limit person-to-person disease transfer [58]. High levels of TNF-α suggests a poor prognosis in TB because it is associated with necrosis and dysregulated granuloma formation. TNF-α is required for granuloma formation in order to contain and/or remove the antigen but its continued presence results in the development of immunopathology as reviewed by Dheda *et al* [58]. IL-4 may present an integral feature of immune regulation as a pure Th1 response is not necessarily beneficial during granulomatous disease but instead the Th1/Th2 ratio should be finely balanced depending on the stage of disease. An early Th1 response is necessary to clear the antigen during the acute stage of infection whereas during chronic disease an abrogation of the Th1 response is necessary to minimise tissue damage caused by necrosis and fibrosis.

In conclusion, this study reports on the expression of Th1 and Th2 immune phenotypes at the site of disease in TB pleuritis. It is shown that HIV positivity results in increased necrosis associated with elevated TNF-α mRNA expression. Future studies should also investigate the expression of other cytokines, chemokines and their receptors to allow a more comprehensive understanding of factors that determine the fine balance between protective and immunopathological responses after infection with *Mtb*.
